# Modeling for the Stringency of Lock-Down Policies: Effects of Macroeconomic and Healthcare Variables in Response to the COVID-19 Pandemic

**DOI:** 10.3389/fpubh.2022.872704

**Published:** 2022-05-25

**Authors:** Giunio Santini, Mario Fordellone, Silvia Boffo, Simona Signoriello, Danila De Vito, Paolo Chiodini

**Affiliations:** ^1^Parliamentary Assembly of the Mediterranean Naples, Naples, Italy; ^2^Mental and Physical Health and Preventive Medicine, University of Campania “Luigi Vanvitelli,” Naples, Italy; ^3^Department of Biology, College of Science and Technology, Temple University, Philadelphia, PA, United States; ^4^Department of Basic Medical Sciences, Neurosciences and Sense Organs, University of Bari “Aldo Moro,” Bari, Italy

**Keywords:** lock-down modeling, socio economic impact, containment policies, COVID-19, SARS-CoV-2 pandemic, healthcare services

## Abstract

**Background:**

The spread of COVID-19 has been characterized by unprecedented global lock-downs. Although, the extent of containment policies cannot be explained only through epidemic data. Previous studies already focused on the relationship between the economy and healthcare, focusing on the impact of diseases in countries with a precarious economic situation. However, the pandemic caused by SARS-CoV-2 drew most countries of the world into a precarious economic situation mostly caused by the global and local lock-downs policies.

**Methods:**

A discriminant analysis performed *via* partial least squares procedure was applied to evaluate the impact of economic and healthcare variables on the containment measures adopted by 39 countries. To collect the input variables (macroeconomic, healthcare, and medical services), we relied on official databases of international organizations, such as The World Bank and WHO.

**Results:**

The stringency lock-down policies could not only be influenced by the epidemical data, but also by previous features of the selected countries, such as economic and healthcare conditions.

**Conclusions:**

Indeed, economic and healthcare variables also contributed to shaping the implemented lock-down policies.

## Introduction

Infectious diseases have become a concerning matter during the last decades, underlining the need for worldwide healthcare systems to prepare effectively in outbreak circumstances and prevent economic and human losses resulting from pandemics (1). Looking back at previous pandemics, the control strategies of the spread of infection focused on the immunization of the population, with an evolution from natural immunization to vaccine-induced immunity. The introduction of mass vaccination and routine pediatric vaccination helped prevent infectious outbreaks. A strong commitment to a wider distribution and acceptance of vaccinations could have avoided the damage suffered by the world population due to COVID-19 (2, 3). Accordingly, some researchers predicted the difficulty of managing the spread of infectious diseases and the potential for significant pandemics due to globalization and the related increase of exchanges among populations and overpopulation (4). There is previous evidence of a strong correlation between income inequalities and the control on the spread of infectious diseases. Specifically, researchers found clear evidence that pandemic events could have devastating effects on the economy and sanitary system of developing countries due to their poor level of preparedness for outbreaks. Precarious public health infrastructures, scarce drug availability, and poor health status are likely to create a fertile ground for the spread of epidemics (5, 6). Indeed, low-income portions of the population are more likely to develop severe symptoms due to their poor health status and experience the negative economic impact of lock-down, such as job losses and income decline (7). Therefore, public health planners need to account for social justice principles when preventing or tackling epidemics and include the protection of underserved or marginalized communities that pose a risk to the entire population (8).

The unintended effects of government decisions about lock-down can raise health risks and a serious threat is a possible negative impact of the pandemic on the diagnosis and follow-up of patients with non-communicable diseases (NCD), with the risk of a delay in screening and treatments due to restricted access to primary health services (9). Another consequence on public health may be a delay in vaccination programs for infections from different pathogens from SARS-COV-2, with great variability depending on the country of interest (10). A recent example is the Ebola outbreak in West Africa, which shifted the public health attention from vaccine-preventable diseases to the new pathogen (11).

Moreover, in most developed countries, higher income per capita translates into higher life expectancy and an overall better health condition of the individual (better nourished and informed) (12). While some researchers state that income growth produces a strong-lowering impact on the mortality rate, others affirm that this correlation is weak, and that mortality has lowered also in countries that remained relatively poor (13). That is due to inequalities in income distribution, which also relates to measures of violence (14). Therefore, the outbreak of infectious diseases must be one of the major concerns for countries with high income per capita but unequal income distribution.

The scientific community has also found evidence of a strong correlation between the health status of a population and some macroeconomic measures. Recent studies have introduced management strategies to prevent and treat COVID-19 assessing the availability of medicines and personal protective equipment (PPE). Their related changes in consumption and the monitoring of prices represent dramatic consequences for families. Patient organizations and pharmacies have a key role in prevention measures and can give suggestions for the authorities for the beginning of pandemics (15). However, in the case of infectious diseases prior to Covid-19, researchers focused more on the economic translation of human loss due to fatalities (16). On the contrary, the global spread of COVID-19 and its medical characteristics are causing an unprecedented worldwide lock-down, whose outcome is difficult to predict while this could deteriorate already precarious contexts and cause crises in apparently stable systems; on the other hand, it has improved educational institutions in some countries, adopting a variety of multiple strategies (17).

Therefore, there is a strong need to understand the main factors affecting lock-down measures. Accordingly, this would allow policymakers to identify the factors needing reinforcement to reduce the impact of pandemics and prevent future economic depressions.

The SARS-CoV-2 pandemic has recalled the constant threat that viruses represent to global health. If we compare the numbers of the affected people by SARS-CoV-2 with those of the previous epidemics of the past two decades, what stands out is a sharp difference in terms of the spread of the disease (18). Indeed, in 2003, the severe acute respiratory syndrome (SARS) caused 8,273 cases and 775 deaths. In 2013, the Middle East respiratory syndrome (MERS) was responsible for 1,139 cases and 431 deaths (19).

Despite the recent outbreak of SARS-Cov-2, researchers have found possible explanations of the lower fatality rate of SARS-CoV-2 compared to those of MERS and SARS, which is strictly related to a wider spread of the disease impacting the host less decisively (20–22).

The infectiousness and high transmissibility of the SARS-CoV-2 pandemic contributed to hardening the challenge for policymakers worldwide and leading to the introduction of social distancing ranging up to full lock-down. Most mitigation strategies included travel restrictions, mandatory quarantine, closures of public spaces, and changes in public health policy. Unfortunately, the heterogeneity of the adopted restriction and the specific political and socio-economical context of each country have made it hard to compare the overall efficacy of global response.

Since a clear overall strategy for the management of the COVID-19 pandemic is lacking, models for the most effective interventions to reduce the spread of the virus are still widely discussed. The aim of this study is to identify the factors that can significantly influence the restriction measures adopted by the governments of 39 countries in response to the COVID-19 pandemic. We decided to take into account the early stages of the pandemic, because this study intends to examine the situation prior to the formation of a global consensus on the application of lock-down measures, analyzing the influence of the input variables on the stringency index. Thus, analyzing what factors could have pushed national leaders toward stronger or weaker lock-down. We assume that the economic and healthcare conditions characterizing a country could be as relevant as the epidemiological indexes in shaping the extent of lock-down measures.

## Materials and Methods

### Countries of Interest and Government Response Stringency Index

For this study, we selected a total of 39 countries, which accounted for more than 70% of global confirmed infections by SARS-Cov-2, as of 1 June 2020. The sample countries are representative of all the major worldwide regions, reflecting the pandemic character of the spread of the disease. We adopted the database of Worldometers as a reference for COVID-19 epidemiologic data (23). The reliability of this database is certified by John Hopkins CSSE and American Library Association (ALA) (24). To assess the extent and the level of lock-down reached by the countries, we have referred to the Government Response Stringency Index on the first day of lock-down, calculated by Oxford University and presented by Our World in Data (25).

### Datasets of Pre-pandemic Characteristics

We analyzed the pre-pandemic characteristics of the countries of interest. We adopted 2018 as a reference for macroeconomic and healthcare data, to prevent any chronological bias, as the date of the most recent update made for all the 39 countries included in this study. To assess the economic and societal features characterizing each country, we selected macroeconomic indicators and related reliable indexes based on relevance for the study and availability of data: Gini coefficient (Gini), percentage of unemployment out of total labor force (unemployment), gross domestic product per capita (GDP), military expenditure, and final consumption expenditure were collected from the online databases of The World Bank. The Human Development Index (HDI) was collected from the database of the United Nations Development Program; we relied on the KPMG database for data on the average of personal income taxes. To include overall indexes of the economic conditions of selected countries, we incorporated the Legatum Prosperity Index (LPI) from the database of the Legatum Institute.

To better understand the resilience of healthcare services when facing a pandemic, we selected other variables that could be explanatory of the size of healthcare coverage and representative of the medical profile of the countries. Data related to the number of hospital beds (per 1,000 population), number of physicians (per 1,000 population), current health expenditure (% GDP), and out-of-pocket expenditure (% current health expenditure) were collected from the databases of The World Bank. Data about the overall performance of healthcare systems (healthcare efficiency), population size (in thousands), population median-age, percentage of current health expenditure as government financing arrangements (GFA), health worker density (hwd), and the Universal Health Coverage (UHC) index were collected from the Global Health Observatory of WHO. Lastly, we included the three medical variables with the highest incidence in the selected countries; data for ischemic heart disease (IHD), chronic obstructive pulmonary disease (COPD), and malignant neoplasms were collected from the Global Health Data Exchange (GHDx), which is the world's most comprehensive catalog of surveys, censuses, vital statistics, and other health-related data of the Institute for Health Metrics and Evaluation (IHME) (26). The medical indicators from GHDx are comprehensive of all ages, both genders, and adjusted for the DALYs of 2017 (Disability-Adjusted Life Years). All the selected variables are reported in Table 1.

**Table 1:** Selected variables and sources.

**Variables**	**Abbreviations**	**Source, Year**
Gini coefficient	Gini	The World Bank, 2018
Percentage of unemployed out of total labor force	Unemployment	The World Bank, 2018
Human Development Index	Hdi	United Nations Development Programme, 2018
Gross Domestic Product (GDP) per capita PPP (USD)	Gdp	The World Bank, 2018
Legatum Prosperity Index	Lpi	Legatum Institute, 2019
Proportion of population living under the line of 1.90 per day (%)	Poverty	World Health Organization, 2018
Military expenditure (% GDP)	military expenditure	The World Bank, 2018
Average personal income tax rate (% of total income)	personal income	KPMG, 2018
Final consumption expenditure (% GDP)	consumption expenditure	The World Bank, 2018
Labor share (% GDP)	labor share	Sustainable Development Goals Data, 2018
Hospital beds (per 1,000 population)	hospital beds	The World Bank, 2018
Physicians (per 1,000 population)	Physicians	The World Bank, 2018
Current health expenditure (% GDP)	health expenditure	The World Bank, 2018
Out-of-pocket expenditure (% current health expenditure)	oop expenditure	The World Bank, 2018
Overall performance of healthcare system	healthcare efficiency	World Health Organization, 2018
Population size (in thousands)	Population	World Health Organization, 2018
Government Financing Arrangements (% current health expenditure)	Gfa	World Health Organization, 2018
Health worker density (per 10,000 population)	Hwd	World Health Organization, 2018
Universal Health Coverage (UHC) index	Uhc	World Health Organization, 2018
IHD: Ischemic Heart Disease (% of total DALYs^a^)	Ihd	GHDx, 2017
COPD: Chronic Obstructive Pulmonary Disease (% of total DALYs^a^)	Copd	GHDx, 2017
Malignant neoplasms (per 100,000 population)	Malignancy	GHDx, 2017
Population median age (years)	population age	World Health Organization, 2018

a *DALYs, disability-adjusted life years*.

*List of variables used to define the profile of each state included in the study. For each variable, the source and the year of the last update are reported. Medical data from WHO refers to the last update available for each variable (2018)*.

### Statistical Analysis

The Government Response Stringency Index was categorized in three stringency groups defined by tertiles of the stringency index distribution that means stringency index was less than the 33rd percentile in the low group, from the 33rd percentile to the 67th percentile in the medium group and higher than the 67th percentile in the high group. The transformation of the stringency index from continuous to categorical variable has been useful in order to obtain classes of countries by “level of stringency” to take into account as outcome variable for a supervised statistical approach of dimensionality reduction.

Variables were reported as median and interquartile range [IQR] and compared at different levels of stringency index by Kruskal-Wallis test.

A discriminant analysis (DA) performed *via* partial least squares (PLS) procedure was applied (i.e., a PLS-DA model) to identify a set of latent dimensions (i.e., components) which explain well the three groups of stringency degree. Notice that first competitors' statistical methods of PLS-DA are principal component analysis (PCA) and factorial analysis (FA). The choice of using the PLS-DA is inspired from the necessity to obtain first a dimensionality reduction, subsequently a groups classification. PLS-DA is very useful in this case because it does not apply separate dimensionality reduction and classification, but it simultaneously detects the best latent subspace to identify the best partition of statistical units. In other words, PLS-DA approach (unlike PCA and factorial FA) maximizes the explained variance constrained by the relationship between latent components and category groups, a priori specified (27, 28). PLS-DA is a variant of Partial Least Squares Regression (PLS-R) that can be used when the response variable is categorical. In fact, PLS-DA is a versatile algorithm that can be used for predictive and descriptive modeling as well as for discriminative variable selection when the matrix of predictors has more variables than observations, and when there is multicolinearity among variables.

The principal outputs of this statistical model consist in (i) a set of latent scores (i.e., components) that are defined as a linear combination of the original variables projected in a new subspace and in (ii) a loadings matrix to define the relationships among the variables and the components. Then, both scores and loadings will define which variables contribute more to the class prediction pre-specified by the response variable.

In order to obtain a variables selection rule and then to define which variables have the biggest contribution on the class prediction, a 0.6 (i.e., 60%) minimum cut-off on the relative contribution was used. The relative contribution is defined as the squared loadings divided by the sum of squared value of all the loadings (notice that each variable will have a loading/contribution for each component).

The optimal number of components was selected using the approach of the maximization of explained variance. Data were analyzed using R software version 3.6.3 (R Foundation for Statistical Computing).

## Results

The median value of the stringency index measured on all 39 selected countries was equal to 81.02 (IQR: 67.36-84.26). The 33rd and the 67th percentile of stringency index distribution were equal to 69 and 82 respectively. A low stringency (from 0 to 69) was adopted by 13 countries, a medium stringency (from 69 to 82) was adopted by 15 countries, and a high stringency (from 82 to100) was adopted by 11 countries.

The stringency index of the 39 selected countries and the corresponding group of stringency index are reported in Supplementary Table 1.

The statistical description of the variables for all countries according to the stringency group is reported in Table 2. The table shows significant relationships between the observed variables and the categorized stringency index. This means that the factors/components identified by the PLS-DA model will be a good synthesis of the observed variables best connected to the used stringency classes.

**Table 2:** Statistical description of the variables for all countries according to the stringency group.

	**Total** **(*n* = 39)**	**Stringency groups**			***p*-value^**a**^**
		**Low** **(*n* = 13)**	**Medium** **(*n* = 15)**	**High** **(*n* = 11)**	
Gini	−0.12 [−0.66, 0.45]	−0.54 [−0.82, −0.12]	0.022 [ −0.42, 0.53]	−0.0040 [−0.33, 1.1]	**0.010**
Unemployment	−0.35 [−0.55, 0.10]	−0.48 [−0.61, −0.23]	−0.40 [−0.55, 0.051]	0.41 [−0.39, 1.3]	**0.007**
Hdi	0.38 [−0.36, 0.76]	0.82 [0.70, 0.90]	0.048 [−0.30, 0.48]	0.035 [−1.3, 0.41]	**0.001**
Gdp	−0.079 [−0.63, 0.58]	0.58 [0.33, 0.74]	−0.28 [−0.59, 0.12]	−0.60 [−1.3, 0.051]	**0.003**
Lpi	0.25 [−0.62, 0.90]	0.92 [0.59, 1.1]	−0.096 [−0.40, 0.42]	−0.55 [−1.7, 0.34]	**0.001**
Poverty	−0.28 [−0.35, −0.11]	−0.31 [−0.35, −0.18]	−0.28 [−0.33,−0.23]	−0.14 [−0.36, 0.36]	0.072
Consumption Expenditure	0.14 [−0.78, 0.61]	0.47 [ 0.36, 1.4]	−0.35 [−0.92, 0.17]	0.059 [−0.50, 0.29]	**0.002**
Military Expenditure	−0.27 [−0.69, 0.46]	−0.37 [−0.69, −0.16]	0.25 [−0.58, 0.46]	−0.27 [−0.74, 0.88]	0.052
Labor share	0.24 [−0.44, 0.69]	0.40 [−0.0030, 0.80]	0.039 [−0.65, 0.51]	−0.0090 [−0.40, 0.71]	0.060
Personal income	0.45 [−0.39, 0.70]	0.45 [−0.22, 1.2]	0.028 [−0.52, 0.49]	0.45 [−0.18, 0.57]	**0.039**
Hospital beds	3.4 [2.9, 5.6]	3.3 [3.0, 4.2]	4.1 [3.1, 5.6]	3.4 [2.3, 5.4]	0.084
Physicians	3.3 [2.7, 4.3]	3.8 [3.1, 4.3]	3.2 [2.4, 3.9]	3.2 [2.6, 4.2]	0.056
Health expenditure	8.3 [6.7, 10]	10 [9.2, 11]	7.2 [6.0, 9.0]	8.0 [7.2, 8.6]	**0.003**
Oop expenditure	18 [14, 27]	14 [13, 18]	24 [18, 31]	19 [12, 29]	**0.008**
Healthcare Efficiency	0.88 [0.77, 0.93]	0.88 [0.86, 0.93]	0.87 [0.74, 0.92]	0.91 [0.73, 0.95]	0.075
Population	17,000 [7,000, 66,000]	17,000 [5,700, 83,000]	11,000 [8,400, 63,000]	47,000 [6,800, 62,000]	0.095
Gfa	22 [8.8, 65]	62 [8.5, 82]	18 [10, 23]	23 [5.4, 36]	**0.019**
Hwd	27 [7.9, 75]	41 [8.3, 120]	27 [8.1, 60]	22 [9.8, 46]	**0.037**
Uhc	79 [75, 83]	84 [81, 86]	79 [75, 83]	76 [75, 79]	**0.001**
Ihd	−0.34 [−0.64, 0.15]	−0.18 [−0.62, 0.11]	−0.38 [−0.66, 0.51]	−0.34 [−0.59, 0.044]	0.098
Copd	0.068 [−0.71, 0.75]	0.41 [−0.21, 0.96]	−0.23 [−0.83, 0.56]	−0.35 [−0.68, 0.52]	0.052
Malignancy	0.28 [−0.81, 0.69]	0.54 [0.28, 1.1]	−0.024 [−0.60, 0.47]	−0.82 [−1.5, 0.37]	**0.003**
Population age	0.40 [−0.46, 0.70]	0.49 [−0.094, 0.72]	0.26 [−0.49, 0.61]	0.071 [−1.7, 0.73]	0.054

a *Kruskal-Wallis test*.

*Data are reported as median [IQR]. Variables with significant difference between groups (at p < 0.05 level) are highlighted in bold*.

As a first step of the PLS-DA approach, we determined the optimal number of components to explain the largest possible proportion of the total variance to guarantee the best model estimation. The optimal number of components is 3, as the three selected components represent 56.85% of the total variance. This is because there is not a significant increase of the explained variance for a number of components bigger than three (Supplementary Figure 1).

**Figure 1:**
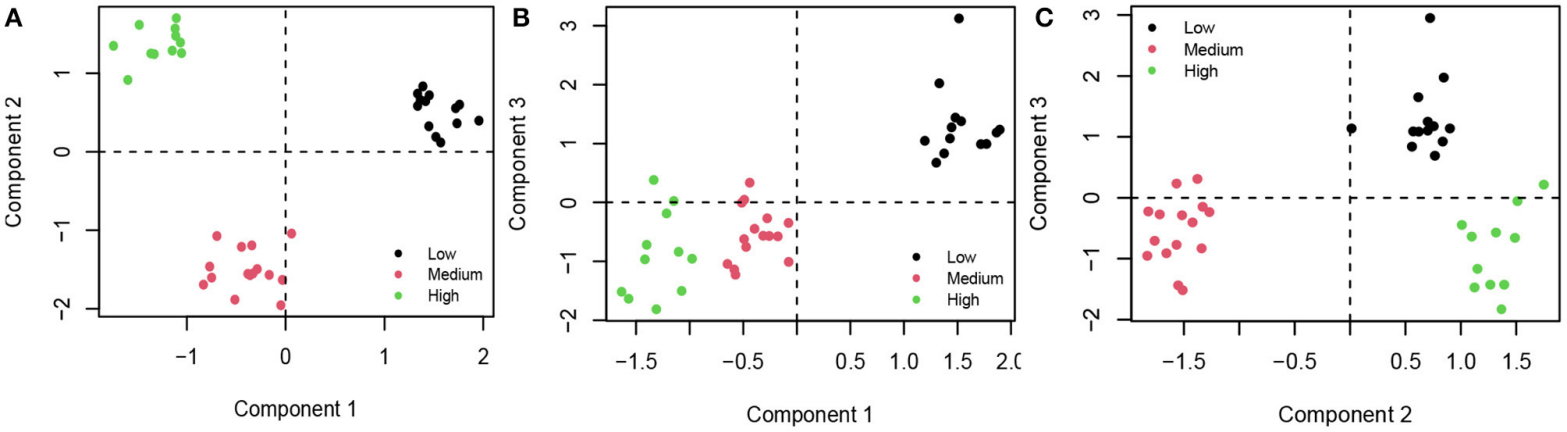
**(A)** Group distribution of the first and the second components; **(B)** Group distribution of the first and the third components; **(C)** Group distribution of the second and the third components. Black, countries that adopted Low Stringency Index; red, countries that adopted Medium Stringency Index; green, countries with High Stringency Index.

Then, we analyzed the distribution of three groups of countries (low, medium, high) for the first three components (Figure 1).

In terms of group distribution, Components 1 and 3 show a positive effect toward the low degree of stringency, while Component 2 shows a positive effect toward the high degree of stringency index; finally, both Components 1 and 3 show a negative effect toward the medium degree of stringency index.

Supplementary Figures 2–4 show the relative contribution of the variables with respect to the corresponding with component 1, 2, and 3, respectively.

In light of the above, the model identified four explanatory variables for Component 1, five for Component 2, and 6 for Component 3.

In the first Component, one variable (Gini coefficient) is negatively correlated, while the remaining three (HDI, LPI, and malignant neoplasms) are positively correlated. Specifically, Gini is a coefficient going from 0 to 1, where 0 is the condition of perfect equality in wealth distribution in an economic system and 1 is maximum inequality. More precisely, the Gini index is twice the area between the Lorenz curve and the curve of perfect equitability. The Lorenz curve for a resource Q is the curve y = L(p), where the Q-poorest fraction p of the population has a fraction L(p) of the whole. Therefore, in a condition of perfect equality, the Lorenz curve coincides perfectly with the curve of perfect equability, and each fraction p of the population has the same fraction L(p) of the whole. This coefficient is key in our work because it efficiently explains wealth inequality, which represents one of the bases of our hypothesis (29).

The HDI is arithmetically calculated to represent the development of a country based on life expectancy, education, and per capita income indicators. The LPI is an index calculated on a set of 12 pillars, which define the prosperity of a country in terms of economy, safety, health, environment, and education. Malignant Neoplasms include all the chronic proliferative diseases diagnosed per 100,000 population, which tend to be higher in developing countries (30, 31).

In the second Component, three variables have a positive correlation (percentage of people living with <1.90% per day, the final consumption expenditure (%GDP), and the average personal income tax rate), and two variables are negatively correlated: out-of-pocket expenditure, which represent the proportion of health expenditure directly paid by households, and ischemic heart disease, one of the leading causes of death globally (32)^1^,^2^. The higher the total Component 2 for each country, the lower the stringency index will be.

The third Component is negatively affected by Labor share (% GDP), hospital beds (per 1,000 population), physicians (per 1,000 population), the overall performance of the healthcare system, positively impacted by the number of the population, and the incidence of COPD, a chronic disease that causes obstructed airflow from the lungs. The higher the number of subjects suffering from chronic inflammatory diseases, the more it affects Component 3, resulting in a greater stringency in cases of lock-downs. In terms of group distribution, Components 1 and 3 show a positive contribution toward the low degree of stringency index, while Component 2 shows a positive contribution toward the high degree of stringency index; finally, both Components 1 and 3 show a negative contribution toward the medium degree of stringency index.

**Figure 2:**
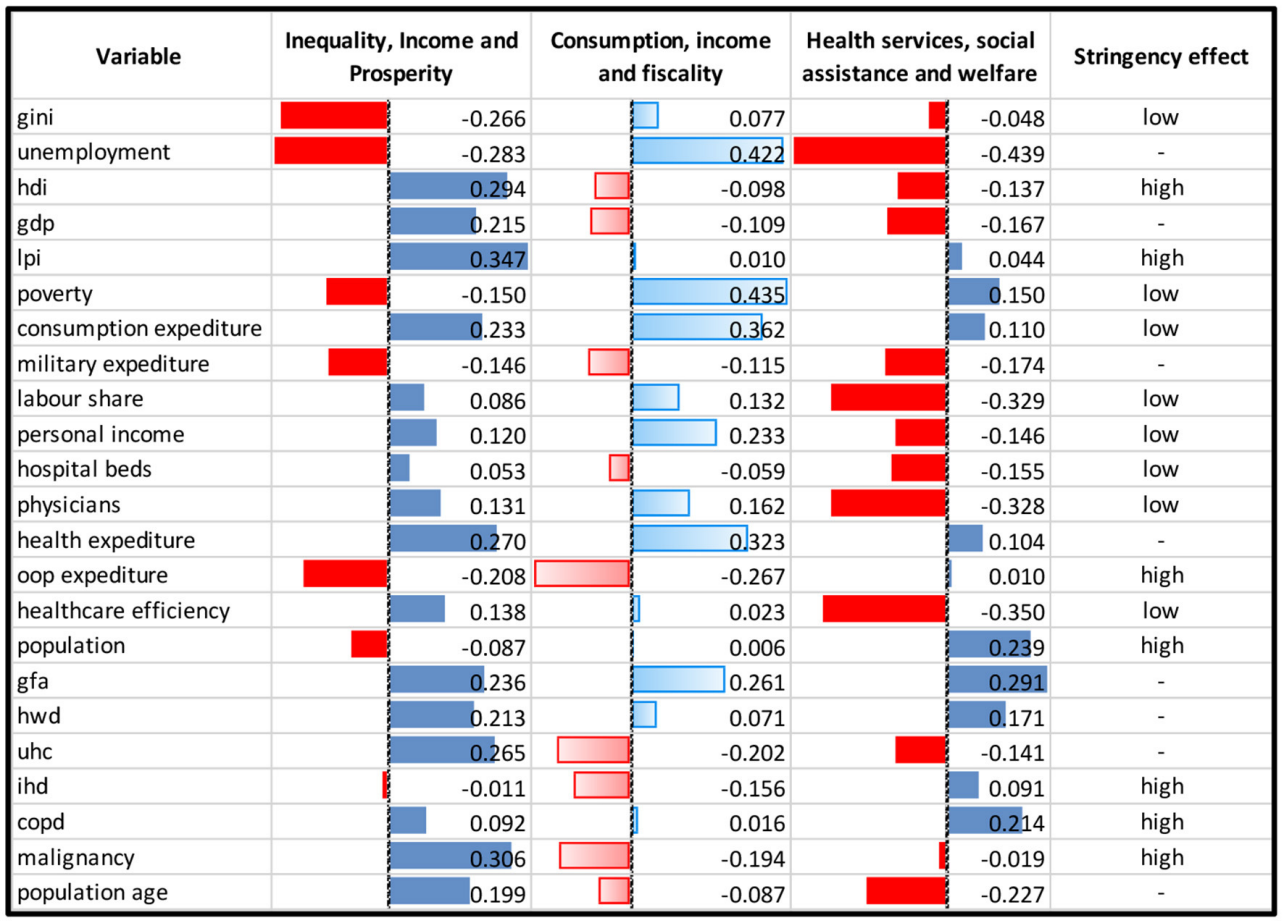
Observed **c**ontribution on components obtained by PLS-DA.

These results can be summarized in Figure 2 in which the following was shown: the contributions measured between variables (listed in the first column) and each component named inequality, income and prosperity, consumption, income and fiscality and health services, social assistance and welfare, respectively. In the last column, the effect that variables have on the stringency index was reported.

Where “high” indicates an increased effect, “low” indicates a decreased effect, and “-” indicates variables not selected by the PLS-DA model. The colors red and blue indicate, respectively, negative and positive relationships between the variables and corresponding components.

## Discussion

This research aimed to investigate the correlation between economic and sanitary characteristics of the sample countries and the extent of the lock-down policies implemented by policymakers. We assumed that the stringency of lock-down policies could not only be influenced by the epidemical data, but also by previous features of the selected countries.

Specifically, this study describes the significant role that **three** main components played in different levels of lock-down stringency adopted by the governments of 39 countries in response to the COVID-19 pandemic.

On the basis of the single variables contribution discussed in the Results section, we tried to identify these components as inequality, income and prosperity (PLS-DA-Component 1: where we have variables that could directly affect the extent of the implemented lock-down policies, in particular in terms of inequality and development), consumption, income and fiscality (PLS-DA-Component 2: where we have fiscal variables that show a correlation with the stringency of lock-down policies), and health services, social assistance and welfare (PLS-DA-Component 3: where we have healthcare and welfare indicators that show a relationship with the stringency of lock-down policies).

Component 1 is negatively correlated to the stringency index, meaning that the higher Component 1 is, the higher the stringency index will be.

The variables that have a greater impact on Component 1 are the Human Development Index (HDI), the Legatum Prosperity Index (LPI), the Gini coefficient. HDI represents the development of a country and LPI defines the prosperity of a country going beyond the macroeconomic and satisfaction measures normally adopted for this type of analysis. Indeed, thanks to its multidimensional character, it “seeks to enhance our understanding of global prosperity by investigating all the different drivers that underlie a country's wealth and wellbeing” (30). The Gini coefficient is negatively correlated to Component 1, meaning that a decrease in the index of this coefficient results in an increased figure for the Component. Therefore, Component 1 suggests that income equality, prosperity, and education may impact the degree of lock-down reached by the countries taken into analysis and be interpreted in comparison to the stringency index as negatively correlated.

Regarding Component 2, the results highlight that higher rates of average personal income taxes and a higher proportion of GDP resulting from final consumption expenditure are related to an increased number for Component 2. As Component 2 is positively related to the stringency index, the growth of the **two** variables above entails an increase of the maximum stringency index reached by the countries analyzed. Moreover, Component 2 is negatively affected by the out-of-pocket health expenditure and the DALY percentage of ischemic heart disease. The **first** variable represents the proportion out of the total health expenditure which comes “out-of-pocket,” meaning that is directly paid by households and is not part of any private agreement or social assistance. Therefore, the negative correlation of this variable with Component 2 could be explained by its capacity to depict effectively the capillarity and the size of a healthcare system, and the assistance it provides.

Component 3 is negatively correlated to the stringency index. This result suggests a close correlation between the size of the healthcare system (or its efficiency) to a reduced stringency index in cases of the outbreak of pandemics. Also, the variable regarding the labor income share, as the compensation of employees over total economy GDP multiplied by total employment, is negatively correlated to Component 3. The variables positively correlated to Component 3 are the population size and the percentage of chronic obstructive pulmonary disease (COPD). The greater size of the population could lead to an increased stringency index in cases of lock-down, given the greater difficulties to impose distancing restrictions in a population where an attempt is made to limit the disease by acting with a rapid diagnosis capable of detecting even asymptomatic subjects (33, 34). COPD is a chronic disease that causes obstructed airflow from the lungs (35). It is usually progressive and is associated with a state of bronchitis and emphysema histo phlogosis, which causes a reduction in respiratory capacity. The higher the number of subjects suffering from chronic inflammatory diseases, the more it affects Component 3, resulting in a greater stringency in cases of lock-down.

Moreover, the timing of the introduction of lock-down measures strongly affects mortality and infecting rates. This study has analyzed the situation as it was as on 1 June 2020, prior to the global consensus around lockdown measures, when countries showed strong divergence in their lockdown policies due to different approaches to the disease, which was changing each day. According to our approach, the stringency of lockdown measures could be explained through macroeconomic features, such as inequality and development. This, combined with the lack of robust reporting systems, may have influenced the approach of other countries, which have applied aggressive lockdown measures despite low prevalence and mortality rates (36).

In the case of other infectious pathologies, the scientists focused their attention mainly on the economic fallout deriving from human losses. On the contrary, the global SARS-CoV-2 pandemic has induced a lock down with consequences that are not easily foreseeable, deteriorating already precarious contexts and improving educational institutions in some countries by adopting multiple strategies.

Finally, we are aware that our proposal has some limitations. Firstly, due to the exploratory nature of the statistical methodology, we can only generate hypotheses to try to give a rational explanation to the phenomenon of interest. However, we think that a good exploratory analysis could help to better understand some mechanisms directly related to lock-down decisions. Secondly, the main approach of this work is the use of a government lock-down stringency index from a single point of time, while in many countries, decisions regarding lock-down rules were changing each day. In this case, we planned a retrospective analysis assuming that there were no differences of the lock-down rules over time to demonstrate that macroeconomic variables should be included in the analysis to assess the readiness of a country, without limiting to epidemiological, medical, and demographic factors. Further investigations of this matter could focus on case studies on a local basis, which could confirm these findings and integrate them with new examples. Future research would be necessary to try to construct an aggregated indicator of lock-down stringency, taking into account different points of time during the pandemic period. In summary, we think that future research would be a chance to try to construct an aggregated indicator of lock-down stringency, taking into account different points of time during the pandemic period, and provide a different approach to classify the countries. For this purpose, other statistical approaches could be performed to validate the results of this study.

## Conclusion

This study describes the significant role that three main components played in different levels of lock-down stringency adopted by the governments of 39 countries in response to the COVID-19 pandemic. From this basis, the following conclusions can be drawn.

First, macroeconomic features can directly affect the extent of the implemented lock-down policies, in particular in terms of inequality and development. Second, the study on healthcare and fiscal variables also revealed a correlation with the stringency of lock-down policies. Again, this result could be interpreted as an effect of the different fiscal regimes, and the different extent of investments in social and healthcare in thesample countries.

For future pandemics, macroeconomic variables should be included in the analysis to assess the readiness of a country, without limiting to epidemiological, medical, and demographic factors. Stakeholders may then implement these findings, adopting policies to reduce inequality and make healthcare more accessible. Further investigations of this matter could focus on case studies on a local basis, which could confirm these findings and integrate them with new examples. Moreover, future research could rely on more definite data on the epidemics and the related impact on global economies. This would surely allow the researchers to quantify more precisely the effect of the presented variables and to add new findings to this subject.

## Data Availability Statement

Publicly available datasets were analyzed in this study. This data can be found at: https://github.com/mfordellone/Modeling-for-the-stringency-of-lock-down-policies or contained within the article.

## Author Contributions

GS contributed to conception and design of the study and wrote the first draft of the manuscript. GS and SB organized the database. MF performed the statistical analysis. SS and MF wrote sections of the manuscript. All authors contributed to manuscript revision and read and approved the submitted version.

## Conflict of Interest

The authors declare that the research was conducted in the absence of any commercial or financial relationships that could be construed as a potential conflict of interest.

## Publisher's Note

All claims expressed in this article are solely those of the authors and do not necessarily represent those of their affiliated organizations, or those of the publisher, the editors and the reviewers. Any product that may be evaluated in this article, or claim that may be made by its manufacturer, is not guaranteed or endorsed by the publisher.

## Footnotes

^1^ https://www.mckinsey.com/featured-insights/employment-and-growth/a-new-look-at-the-declining-labor-share-of-income-in-the-united-states

^2^ https://www.oecd.org/g20/topics/employment-and-social-policy/Income-inequality-labour-income-share.pdf
