# Supplementary material for: Modeling for the Stringency of Lock-Down Policies: Effects of Macroeconomic and Healthcare Variables in Response to the COVID-19 Pandemic
Source: Front Public Health. 2022 May 25;10:872704. doi: 10.3389/fpubh.2022.872704 (PMC9174749; doi:10.3389/fpubh.2022.872704)
Supplement: Supplementary file 1 [file Data_Sheet_1.PDF]

Supplementary Material

1 Supplementary Data

Dataset is available at the following link https://github.com/mfordellone/Modeling-for-the-stringency-of-lock-down-policies.git

2 Supplementary Figures and Tables

2.1 Supplementary Figures

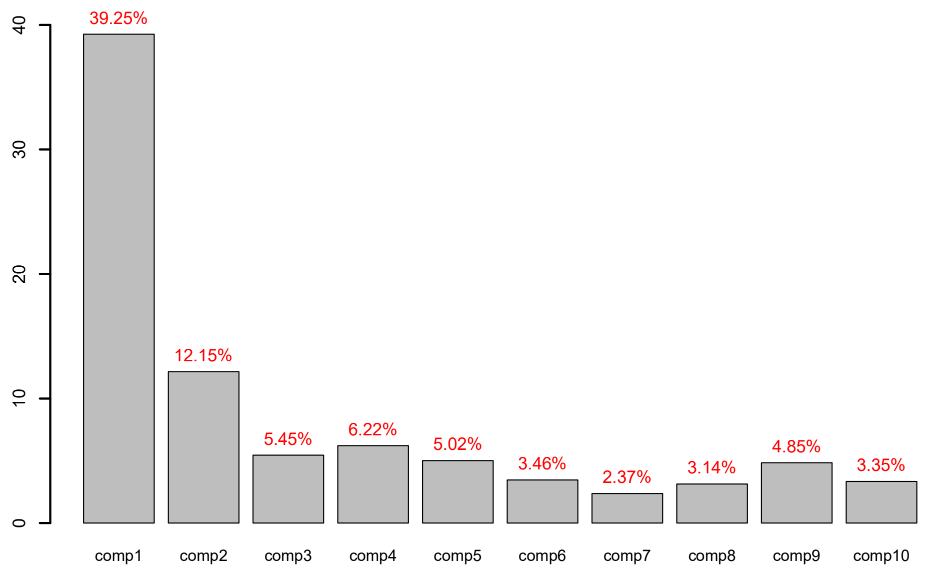

Supplementary Figure 1. Bar-plot of the cumulative variance proportion explained by the first ten components.

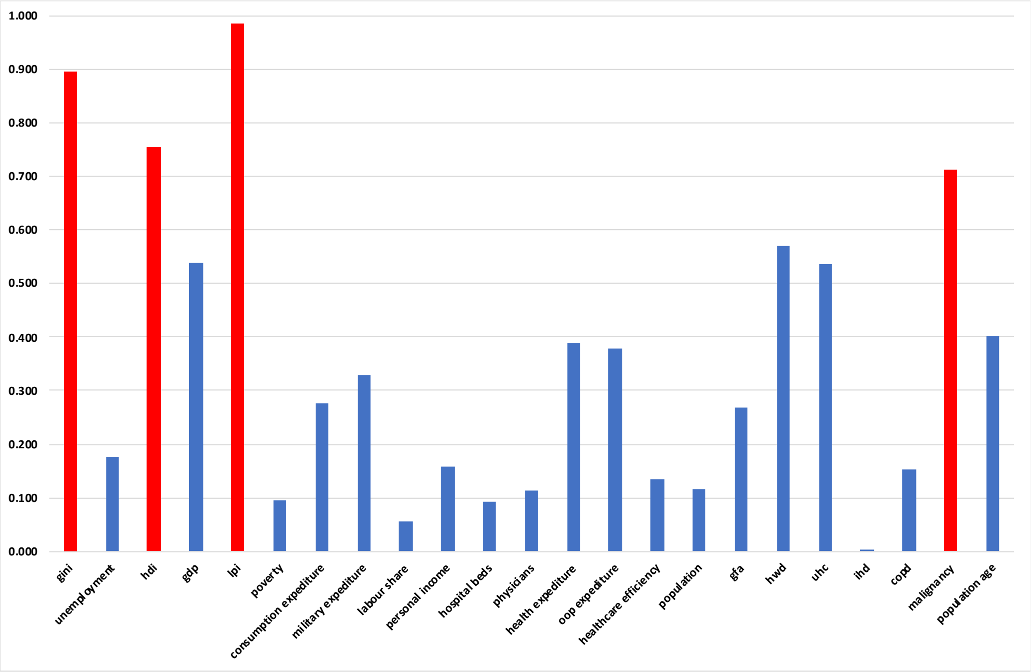

Supplementary Figure 2. Relative contributions of the variables with respect to the Component 1. In blue: variables with a contribution lower than 0.6; in red: variables with a contribution higher than 0.6.

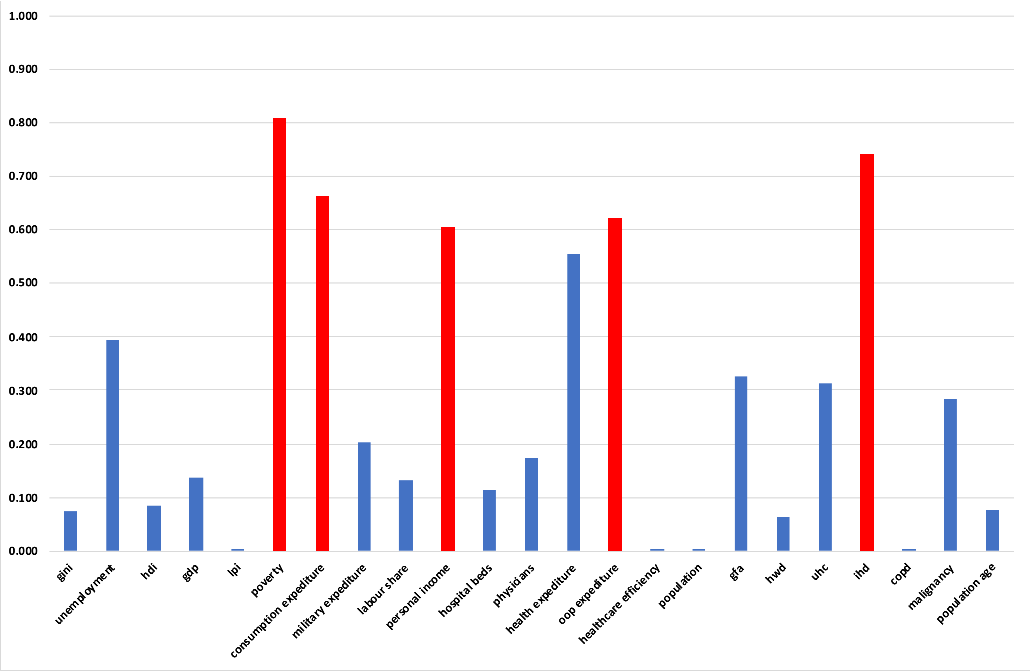

Supplementary Figure 3. Relative contributions of the variables with respect to the Component 2. In blue: variables with a contribution lower than 0.6; in red: variables with a contribution higher than 0.6.

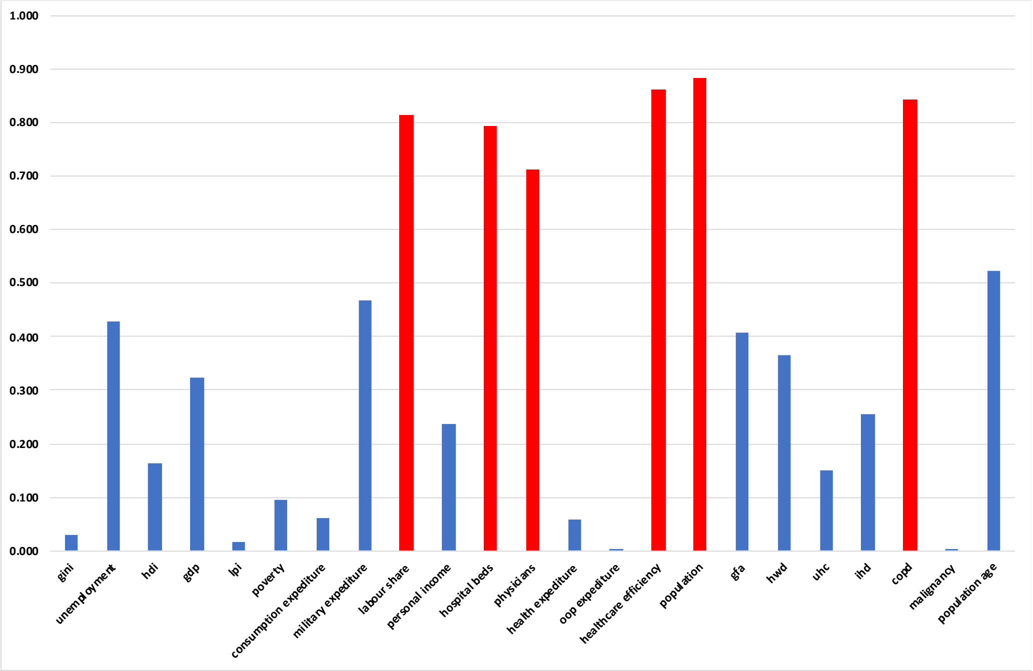

Supplementary Figure 4. Relative contributions of the variables with respect to the Component 3. In blue: variables with a contribution lower than 0.6; in red: variables with a contribution higher than 0.6.

2.2 Supplementary Tables

Supplementary Table 2. Stringency index values (continuous and categorical) for all countries.

Country	Stringency index	Stringency group
Australia	64.35	Low
Austria	85.19	High
Belgium	81.48	Medium
Canada	61.11	Low
Chile	73.15	Medium
China	81.02	Medium
Colombia	87.96	High
Costa Rica	84.26	High
Czech Republic	82.41	Medium
Denmark	62.96	Low
Estonia	80.56	Medium
Finland	62.96	Low
France	90.74	High
Germany	68.06	Low
Greece	84.26	High
Hungary	67.59	Low
Iceland	50.93	Low
India	100.00	High
Indonesia	60.65	Low
Ireland	85.19	High
Israel	81.48	Medium
Italy	82.41	Medium

Japan	43.52	Low
Lithuania	81.48	Medium
Luxembourg	72.22	Medium
Mexico	82.41	Medium
Netherlands	62.04	Low
Norway	67.59	Low
Poland	81.48	Medium
Portugal	82.41	Medium
Russia	87.04	High
Slovenia	89.81	High
South Africa	87.96	High
Spain	85.19	High
Sweden	40.74	Low
Switzerland	76.85	Medium
Turkey	80.56	Medium
United Kingdom	70.37	Medium
United States	67.13	Low
